# Recurrent optic neuritis as the only manifestation of chronic hepatitis B virus flare: a case report

**DOI:** 10.1186/s13256-018-1810-0

**Published:** 2018-10-16

**Authors:** Diana Curras-Martin, Natasha Campbell, Attiya Haroon, Mohammad A. Hossain, Arif Asif

**Affiliations:** Department of Medicine, Jersey Shore University Medical Center, Hackensack Meridian Health, Neptune, NJ 07753 USA

**Keywords:** Optic neuritis, Chronic hepatitis B virus, Autoimmune reaction

## Abstract

**Background:**

Autoimmune reactions have been associated with acute hepatitis B virus infection. Among these optic neuritis is a rare presentation with only a handful of cases reported in the literature thus far. The pathophysiologic mechanism governing this phenomenon includes high levels of circulating immune complexes, tissue deposit, and complement activation cascade.

**Case Presentation:**

In this report, we present the case of a 46-year-old African American man with a past medical history of untreated chronic hepatitis B virus, diagnosed 5 years ago, who presented to our facility on two occasions with the chief compliant of blurry vision. He was diagnosed with optic neuritis associated with acute on chronic hepatitis B virus infection, where the recurrent visual impairment was the main presenting symptom. Because hepatitis constituted a relative contraindication for steroid therapy, our patient was solely treated with antiviral medication. Antiviral therapy resulted in complete resolution of his symptoms and improvement in his liver function.

**Conclusions:**

Further studies are necessary to conclusively establish whether antiviral therapy can be employed as the sole therapy in immune complex-mediated optic neuritis, in the setting of active recurrent hepatitis B infection.

## Background

Autoimmune reactions are commonly encountered with acute hepatitis B virus infection. Vasculitis, glomerulonephritis, arthritis, and optic neuritis have all been described in the literature [1]. High levels of circulating immune complexes, tissue deposit, and complement activation support the pathophysiology [2]. Here, we present a case of recurrent optic neuritis associated with reactivation of chronic hepatitis B virus (HBV) flare, where the ocular symptoms constituted the chief complaint.

## Case presentation

A 46-year-old African American man presented with complaints of progressive, bilateral, blurring (more in his right eye than his left) for the past 5 days. Our patient did not report a history of a prior similar episode. He denied the presence of any associated pain, trauma to his eye, redness of eye, headache, dizziness, weakness/paresthesia, changes in hearing, fever, chills, weight changes, recent travel, insect or tick bite, or sick contact. His past medical history was relevant for hepatitis B virus (HBV) diagnosed 5 years ago, and his past family history was noncontributory.

### Clinical findings

His vital signs on presentation revealed a blood pressure of 132/59 mmHg, heart rate of 72 beats/min, respiratory rate of 18/min, oxygen saturation of 100% at room air, and a temperature of 98.6 °F. On physical examination, our patient was in no apparent distress, awake, alert, and oriented to person, place, and time. He was icteric with palpable nontender hepatomegaly. Heart and lung examinations were unremarkable. A neurological examination revealed significantly reduced visual fields in both eyes with normal pupillary size and reaction, however, a funduscopic examination was unremarkable. His extraocular eye movements were intact without ptosis. Other neurological examinations were normal including motor, sensory, and cranial nerves.

### Timeline

Laboratory values from his first admission, second admission, and follow-up at 6 months were analyzed. On biochemistry, his electrolytes were within normal limits on all three occasions. Transaminase levels were markedly elevated on both the first and second admissions ranging from 500 to 1600 IU/L for aspartate aminotransferase (AST) and from 400 to 1500 IU/L for alanine aminotransferase (ALT). At the time of his 6-month follow-up, his AST and ALT levels were 51 and 87 IU/L respectively. Similarly, both his international normalized ratio (INR) and total bilirubin had improved to a normal range at follow-up. On complete blood count, his platelets remained stable on all three visits ranging from 127 to 210 kU/L. On both the first and second admissions, our patient was found to have positive hepatitis B envelope antigen, which was negative at the 6-month follow-up. Also, the hepatitis B virus deoxyribonucleic acid (DNA) load had markedly decreased from an average of 150 million IU/mL to 5000 IU/mL. Otherwise, his hepatitis B core immunoglobulin M (IgM), surface antibody and antigen, and hepatitis B virus envelope antibody remained unchanged.

### Diagnostic assessment

A presumptive diagnosis of optic neuritis was made and a differential diagnosis included multiple sclerosis and infectious etiology. The laboratory data are summarized above. Other serology test results, including for hepatitis C virus (HCV), hepatitis A virus (HAV), human immunodeficiency virus (HIV), syphilis, babesia and Lyme disease, were negative. A magnetic resonance imaging scan (MRI), with and without gadolinium, of his brain, orbits, neck, and spine were unremarkable. A lumbar puncture was performed considering multiple sclerosis in the differential diagnosis; cerebrospinal fluid (CSF) cytology was negative for infection and malignant cells, but showed few mature lymphocytes admixed with monocytes. CSF isoelectric focusing/immunofixation demonstrated identical bands in the CSF, consistent with a systemic, no intrathecal immune reaction, and was considered to be a negative result for oligoclonal bands. His albumin CSF level was 25 mg/dL, albumin index 6.3, and CSF IgG/albumin ratio 0.26. A diagnosis of retrobulbar optic neuritis was made in association with an HBV flare.

### Therapeutic intervention

During the first admission, our patient was started on prednisone 1 mg/kg/daily for 14 days and entecavir 1 mg/daily long-term therapy.

### Follow-up and outcomes

After few days of steroids, his blurring of vision completely resolved. He was discharged home with follow-up appointments as an outpatient. After 12 months, he presented with similar complaints after stopping his antiviral medication for 2 months. A presumptive diagnosis of optic neuritis associated with acute on chronic HBV was made. Because he had a flare of his viral hepatitis (with an AST level of 1558 IU/L and ALT level of 1488 IU/L), steroid therapy was avoided, and he was treated with entecavir alone. Our patient’s visual acuity improved after 5 days of entecavir (1 mg once daily) therapy. His abdominal tenderness resolved, liver enzymes improved, and his INR returned to baseline level.

## Discussion

Worldwide, 240 million individuals are estimated to have chronic HBV infection. The incidence of HBV in USA is 21,900 annually and approximately 5% of adults become chronically infected [3]. The detection of serum HB surface antigen for more than 6 months is considered to be diagnostic of chronic HBV infection, whereas the decision to treat is based upon the presence of HBe antigen, ALT, and the HBV DNA viral load. Several extrahepatic manifestations are associated with chronic HBV infection, such as arthralgia, vasculitis, neuritis, and membranous and membranoproliferative glomerulonephritis. While our patient had HBV flare with resultant elevation of his AST and ALT levels, his visual abnormality was the only extrahepatic manifestation encountered. With entecavir therapy, the ocular manifestation resolved and his liver function normalized.

Optic neuritis is the primary inflammation of the optic nerve and is often referred to as retrobulbar optic neuritis [4, 5]. Approximately, two thirds of cases demonstrate a normal optic disc on funduscopic examination, while others may demonstrate blurring of the disc. The case presented in this report revealed a normal disc on funduscopic examination, which consequently did not assist us in making the diagnosis. Post-viral optic neuritis usually precedes the infection by 1–3 weeks. Several additional viruses have been associated with this phenomenon including Epstein-Barr, measles, mumps, influenza, and varicella-zoster virus [6]. The use of methylprednisolone 1 mg/kg/daily for 14 days hastens recovery, and is considered a reasonable option according to current guidelines [7, 8]. However, we could not use steroid therapy due to the acute flare of HBV in our patient.

Post-hepatitis B infection polyneuropathy in adults and optic neuritis associated with hepatitis B vaccination, mainly in children, have been reported frequently in the medical literature; however, there are only two cases of post-infectious retrobulbar optic neuritis associated with HBV infection reported in the medical literature so far [9, 10, 11]. The first case, a post-infectious acute HBV optic neuritis was described by Galli *et al.* at the University of Milan in 1986 [9]. The patient (a young woman) reported decreased visual acuity after normalization of liver enzymes that remitted after a course of steroids [9]. The second case was reported by Achiron *et al.* at (Emory University School of Medicine, Atlanta in 1994) a middle-aged woman who developed painful loss of vision and fatigue 1 month after her acute HBV infection subsided. Further investigation of this case revealed the presence of papilledema during the ophthalmologic examination and was associated with glomerulonephritis and arthritis. Ocular symptoms significantly improved after 1 week of steroids [10].

In our case, the initial presentation was treated with steroids at the standard dose of methylprednisolone (1 mg/kg/ daily) for 14 days with good response. The steroid use in the two previously reported patients occurred in the setting of post-viral optic neuritis, whereas the presence of severe active infection during our patient’s second admission constituted a relative contraindication to steroid therapy. However, our patient responded well to the re-initiation of his antiviral regimen without any steroid use.

The most likely pathophysiological mechanism governing this presentation is an elevated number of immune-complex depositions and subsequent complement cascade activation [12]. It has been reported that aggressive forms of chronic hepatitis B virus are characterized by a higher frequency of circulating immune complexes along with higher anti-HBs antibodies [13], increasing the possibilities of immune-related events such as the optic neuritis observed in our patient.

## Conclusions

Retrobulbar optic neuritis in association with HBV is rarely the only manifestation of chronic hepatitis B flare. The recommended standard of care involves the use of systemic steroids. However, given the recurrence of HBV flare, the risk of worsening the hepatitis with a high-dose steroid is a reality. Consequently, our options were limited to antiviral therapy alone. Our patient successfully achieved a complete resolution of symptoms with improvement of his liver function after treatment with entecavir alone. Further studies are needed to establish the role of antiviral therapy alone in the management of optic neuritis encountered in the context of hepatitis flare.
